# Biosynthesis and assembly of the Collagen IV-like protein Pericardin in *Drosophila melanogaster*

**DOI:** 10.1242/bio.030361

**Published:** 2018-04-15

**Authors:** Ariane C. Wilmes, Nora Klinke, Barbara Rotstein, Heiko Meyer, Achim Paululat

**Affiliations:** University of Osnabrück, Biology, Department of Zoology and Developmental Biology, Barbarastraße 11, 49076 Osnabrück, Germany

**Keywords:** Cellular biology, Developmental biology, Pericardin, Extracellular matrix, Cardiac matrix, Cardiogenesis, Dorsal vessel, Heart

## Abstract

In *Drosophila*, formation of the cardiac extracellular matrix (ECM) starts during embryogenesis. Assembly and incorporation of structural proteins such as Collagen IV, Pericardin, and Laminin A, B1, and B2 into the cardiac ECM is critical to the maintenance of heart integrity and functionality and, therefore, to longevity of the animal. The cardiac ECM connects the heart tube with the alary muscles; thus, the ECM contributes to a flexible positioning of the heart within the animal's body. Moreover, the cardiac ECM holds the larval pericardial nephrocytes in close proximity to the heart tube and the inflow tract, which is assumed to be critical to efficient haemolymph clearance. Mutations in either structural ECM constituents or ECM receptors cause breakdown of the ECM network upon ageing, with disconnection of the heart tube from alary muscles becoming apparent at larval stages. Finally, the heart becomes non-functional. Here, we characterised existing and new *pericardin* mutants and investigated biosynthesis, secretion, and assembly of Pericardin in matrices. We identified two new *pericardin* alleles, which turned out to be a null (*pericardin*^3-548^) and a hypomorphic allele (*pericardin*^3-21^). Both mutants could be rescued with a genomic duplication of a fosmid coding for the *pericardin* locus. Biochemical analysis revealed that Pericardin is highly glycosylated and forms redox-dependent multimers. Multimer formation is remarkably reduced in animals deficient for the prolyl-4 hydroxylase cluster at 75D3-4.

## Abstract (precis)

**Summary:** We identified two new *pericardin* alleles. Both mutants could be rescued with a genomic duplication of a fosmid coding for the *pericardin* locus. Biochemical analysis revealed that Pericardin is highly glycosylated and forms redox-dependent multimers.

## INTRODUCTION

By underlying or encasing a multitude of cells or tissues, extracellular matrices (ECMs) are essential to several physiological processes including tissue protection, tissue scaffolding, and cell signalling. Biochemical analysis, which is generally impeded by the insoluble and frequently cross-linked nature of the ECM, has shown that the complexity of matrices is much higher than previously expected. It has been reported that the ‘matrisome’, which collectively encompasses the proteins that constitute the ECM, comprises more than 300 proteins in mammals, including collagens, proteoglycans, growth factors, and receptors (Hynes and Naba, 2012). The complexity of matrices is not only reflected by the number of proteins that constitute the matrix, but also by the different ratio with which the various components contribute and by the appearance of unique components in matrices of specific tissues. For example, while a high amount of Collagen I is characteristic of tendons, basement membranes (BMs) contain large amounts of Collagen IV, Laminins, Perlecan, and other proteins. Due to its diverse physiological function, the ECM is more than a homogeneous mass of proteins and carbohydrates. Within the meshwork of its structural components, the ECM is spatially patterned and thereby provides locally restricted reaction environments and structural micro-compartments.

The *Drosophila* heart is considered as a model for a specialised ECM composition that ensures proper tissue integrity, functionality, and organ performance (Rotstein and Paululat, 2016). In *Drosophila*, at present, only four collagens or collagen-like proteins have been identified. One of these proteins is Pericardin (Prc) (Chartier et al., 2002); the others are Collagen IV alpha2 (Viking, Vkg) (Yasothornsrikul et al., 1997), Cg25c (Dcg1) (Cecchini et al., 1987; Natzle et al., 1982; Yasothornsrikul et al., 1997), and Multiplexin (Adams et al., 2003; Blumberg et al., 1988; Kelemen-Valkony et al., 2012; Monson et al., 1982; Volk et al., 2014; Yasothornsrikul et al., 1997). The Pericardin precursor protein consists of 1713 amino acids and harbours an N-terminal signal peptide as well as a long repeat region separated into a collagen-like domain and a non-collagen-like domain, with the former containing 26 atypical and several typical (Gly-X-Y)*_n_* repeats (Katti et al., 2000). In addition, a single potential Integrin-binding site (RGD) is present at the C-terminus (Chartier et al., 2002; Drechsler et al., 2013; Volk et al., 2014). In contrast to the ubiquitously distributed Collagen IV, Pericardin assembles specifically within distinct matrices: these include the matrix of the heart tube, the surface of pericardial cells and oenocytes, and the cap cells of chordotonal organs. Lack of Pericardin, or its ECM adapter protein Lonely heart (Loh), causes heart failure upon ageing (Drechsler et al., 2013; Rotstein et al., in revision). During development, Pericardin is synthesised and secreted by different tissues: first, during embryogenesis, the pericardial cells secrete Pericardin; later, in first and second instar larvae, the main source of Pericardin secretion is the adipocytes. After biosynthesis, secretion, and release into the haemolymph, Pericardin specifically assembles at the outer surface of the cardiac tube and incorporates into the meshwork formed by typical structural components of basement membranes such as Collagen IV, Perlecan, and Nidogen. Adipocyte-specific knock-down of *Sar1* expression inhibits Pericardin secretion and thereby affects the formation of a proper heart ECM in *Drosophila* (Drechsler et al., 2013; this work). When Pericardin is not expressed, not secreted, or mislocalised, heart integrity is lost, which ultimately results in heart failure and heart collapse. These findings demonstrate that the assembly of a single structural protein, such as Pericardin, in the larval heart is essential for organ integrity and that adipocytes are the major source of distinct ECM components delivered to the heart tube.

Aiming to extend the current knowledge on how the specific meshwork of structural ECM constituents that characterise the heart matrix is established, we investigated aspects of the biosynthesis, secretion and deposition of Pericardin in the cardiac matrix in more detail. The Pericardin protein displays collagen-like features that led to the assumption that Pericardin forms, like Collagen IV, trimeric helices that incorporate into matrices (Chartier et al., 2002; Drechsler et al., 2013).

Thus, we focused particularly on components that are known to play an important role in Collagen IV processing, asking whether these enzymes also process Pericardin. Hydroxylation of proline and lysine residues of collagen proteins, taking place within the ER of the collagen-synthesising cells, leads to dimer- and trimerisation by converting proline or lysine into hydroxyproline or hydroxylysine (Gorres and Raines, 2010; Myllyharju, 2003). This reaction is catalysed by various proteins such as Prolyl 4-hydroxlases (PH4), which map to different loci within the genome. Lysine hydroxylation is performed by Lysyl-hydroxylases of which only one, dPlod, is present in the fly genome. Prolyl 4-hydroxylases are comprised of an α_2_β_2_ tetramer; the β-subunit is encoded, in *Drosophila*, by the *pdi* gene (Pdi, Protein-disulfide isomerase).

We found that Pericardin processing, i.e. multimerisation, is not blocked in mutants for *pdi* and *dplod*, and – to some extent – is inhibited in deficiencies that delete a cluster of PH4-encoding genes, which is in contrast to Collagen IV processing phenotypes seen in mutants for *pdi*, *dplod*, or *PH4* genes (Abrams and Andrew, 2002; Bunt et al., 2011; Kelemen-Valkony et al., 2012; Molnar et al., 2005; Myllyharju and Kivirikko, 2004; Pastor-Pareja and Xu, 2011; Yasothornsrikul et al., 1997). Possibly, redundant or residual activity of the enzymes is sufficient for Pericardin (but not for Collagen IV) maturation and cardiac assembly. Furthermore, our recent results show that Pericardin deposition at the embryonic cardiac matrix is, unlike deposition of Collagen IV (Pastor-Pareja and Xu, 2011), not necessary for the recruitment and incorporation of additional structural ECM proteins such as Laminin, Nidogen, or Perlecan. Our Western blot analyses provide initial evidence that Pericardin forms intermediate dimers as well as multimeres. Like many other secreted matrix proteins, Pericardin is extensively glycosylated, indicating cross-linking of Pericardin with other ECM proteins via carbohydrate chains. Finally, we extended previous analyses of *pericardin* mutant phenotypes by characterising two new EMS-induced *pericardin* alleles, which we identified in a genetic screen for mutants displaying post-embryonic heart malformations. One of the new *pericardin* alleles turned out to be a protein null allele, whereas the other one represents most likely a hypomorphic allele with Pericardin being expressed but misassembled.

## RESULTS

### Identification of two new *pericardin* mutants

**Fig. 1.**
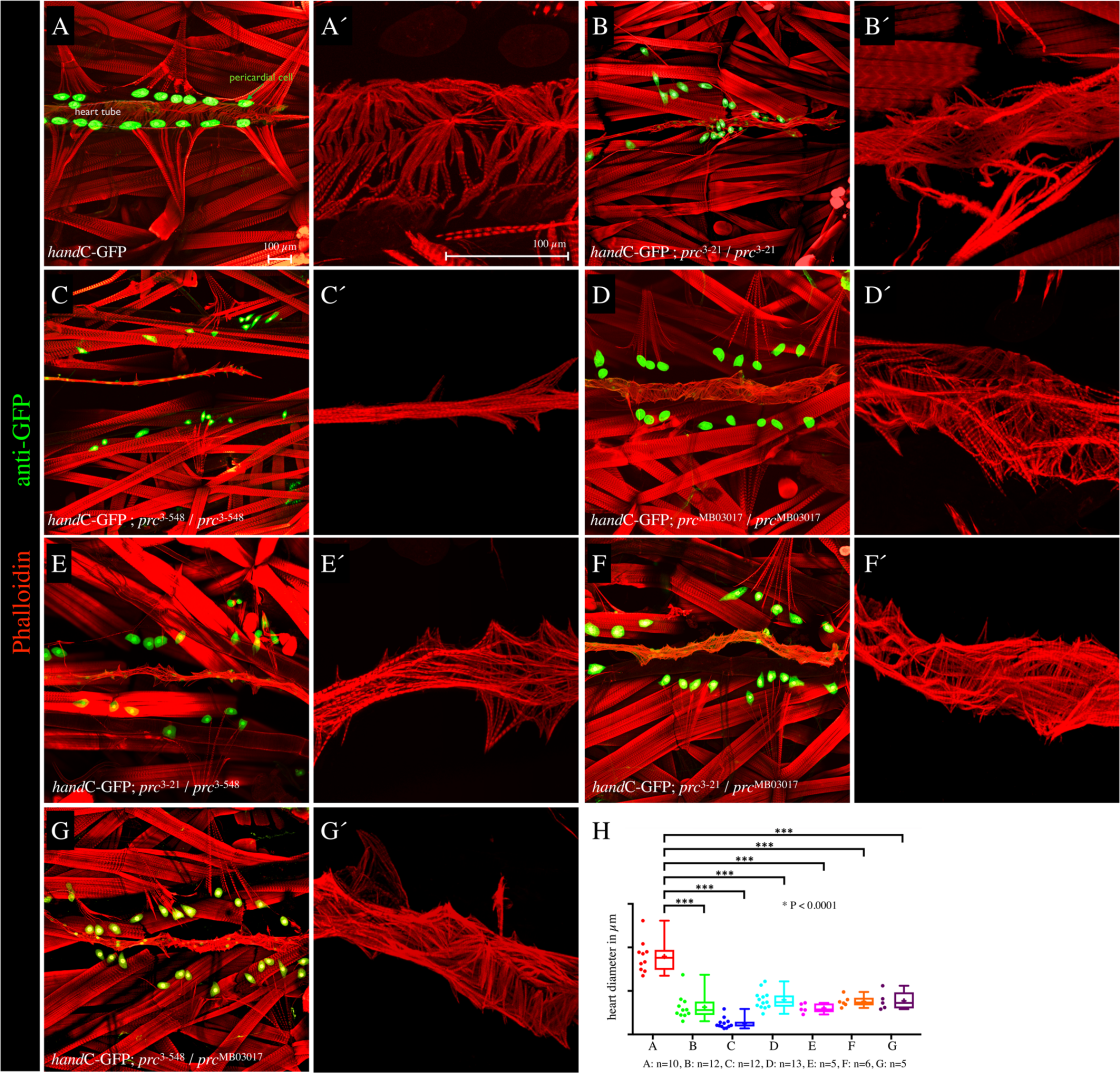
**Cardiac phenotype of *pericardin* alleles.** (A-G′) Dissected third instar larvae were stained with anti-GFP (green channel) to visualise the GFP reporter expression in cardiac cells driven by *hand*-GFP (Sellin et al., 2006) and counterstained with Phalloidin-TRITC to label the actin fibres in cardiomyoblasts and somatic muscles (red channel). Pictures show the heart of dissected third instar larvae with the genotype *hand*C-GFP (A,A′), *hand*C-GFP; *prc*^3-21^/*prc*^3-21^ (B,B′), *hand*C-GFP; *prc*^3-548^/*prc*^3-548^ (C,C′), *hand*C-GFP; *prc*^MB03017^/*prc*^MB03017^ (D,D′), *hand*C-GFP; *prc*^3-21^/*prc*^3-548^ (E,E′), *hand*C-GFP; *prc*^3-21^/*prc*^MB03017^ (F,F′) and *hand*C-GFP; *prc*^3-548^/*prc*^MB03017^ (G,G′). The higher magnifications (red channel) illustrate the orientation of F-actin fibres in the heart tube. (H) For quantification of the observed reduction of the heart diameter, five to 13 individual animals were used to measure the width along the heart chamber (with 10 µm distance between the individual reading points). The heart diameter is significantly reduced compared to wild type (***P≤0.0001, unpaired *t*-test). Higher-magnification images show representative areas of the dorsal vessel with the respective genotype, either taken from the same animal shown on the right side or a different animal with the same genotype. H shows box-and-whisker plots with lower and upper quartiles and the median. Whiskers indicate variability outside the upper and lower quartiles. In addition, scatter plots are shown.

For screening a collection of pupal lethal EMS-induced mutants (Koundakjian et al., 2004), we made use of our previously established cardiac *hand*-GFP reporter to monitor age-related cardiac integrity defects in living animals without the need of dissection (Paululat and Heinisch, 2012; Sellin et al., 2006) (Fig. 1A). This led to the identification of two new mutant alleles carrying EMS-induced mutations on the third chromosome, PMM3-21 (*prc*^3-21^) and PMM3-548 (*prc*^3-548^), with both displaying an irreversible detachment of the pericardial nephrocytes from the heart tube eminent in third instar larvae (Fig. 1B,C). Age-dependent displacement of pericardial nephrocytes is accompanied by structural defects in the cardiac extracellular matrix, a phenotype that resembles the malformations previously observed in mutants for the Pericardin adapter protein Lonely heart, as well as in *pericardin* mutants (Chartier et al., 2002; Drechsler et al., 2013). In addition, we observed severely misguided myofibers in the cardiomyocytes of mutant larvae (Fig. 1B-G), a phenotype previously found in cardiac ECM mutants (Drechsler et al., 2013). In this context, the connection between sarcomeres and the ECM, which is established by Integrins and Dystroglycans, might be lost in ECM mutants, which in turn affects regular organisation and orientation of myofibers (Fig. 1).

Genetic and phenotypic analysis of PMM3-21/PMM3-548 transheterozygous animals revealed that the two alleles fail to complement each other (Fig. 1E). This observation indicates that the same gene is mutated in both lines. A genetic analysis showed that the newly discovered mutants displaying a cardiac ECM phenotype failed to complement the previously characterised *pericardin* transposon allele *pericardin*^MB03017^ (Fig. 1F,G) as well as the *pericardin* deficiency Df(3L)*vin6*. The new *pericardin* alleles were named *pericardin*^3-21^ and *pericardin*^3-548^. Homozygous *pericardin*^MB03017^, *pericardin*^3-21^, *pericardin*^3-548^, and transheterozygous animals with the genotype *pericardin*^3-21^/*pericardin*^MB03017^, *pericardin*^3-548^/*pericardin*^MB03017^, and *pericardin*^3-21^/*pericardin*^3-548^ showed similar phenotypes when third instar larvae were stained for *hand*-GFP to illustrate dissociation of pericardial cells from the heart tube and for F-actin to visualise heart tube collapse (Fig. 1). The latter phenotype was systematically quantified by measuring the diameter of the heart tube every 10 µm along the anterior-posterior axis (heart chamber proper) in control and in mutant animals (Fig. 1H). All *pericardin* alleles (homozygous or transheterozygous conditions) showed a severe heart tube luminal collapse resulting in a significantly reduced heart diameter and a severe F-actin disarrangement, in addition to the detachment of the pericardial nephrocytes from the heart tube.

**Fig. 2.**
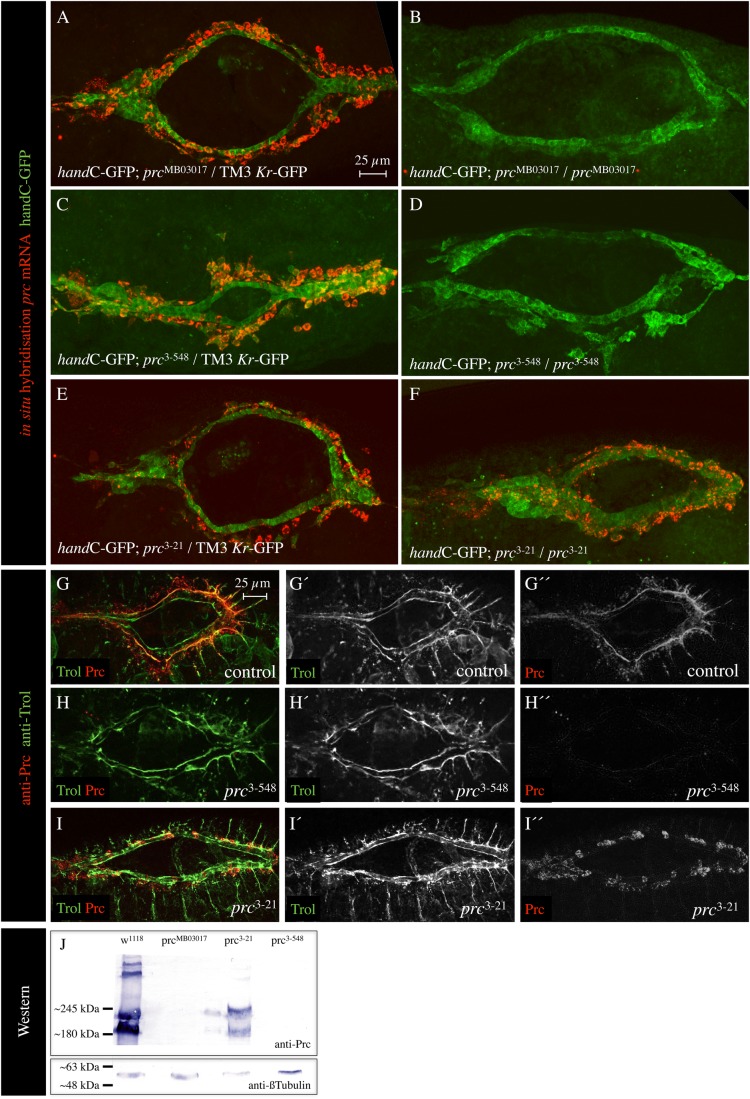
**Characterisation of *pericardin* alleles.** (A-F) Stage 15-16 embryos stained for *prc* transcripts (red channel) and *hand*C-GFP (heart cells, green channel). The *pericardin* mutants with the genotype *hand*C-GFP; *prc*^MB03017^/*prc*^MB03017^ (B), *hand*C-GFP; *prc*^3-548^/*prc*^3-548^ (D) and *hand*C-GFP; *prc*^3-21^/*prc*^3-21^ (F) were probed and compared to the control animals (A,C,E). (G-I) Stage 15-16 embryos stained for Trol/Perlecan (green channel) and Prc protein (red channel). Beside the merged images, single channels are shown individually, as well. (J) Western blot analysis of the respective *prc* mutants is depicted. As a loading control, the blot was probed for anti-β-Tubulin. All protein extracts contain sufficient amounts of proteins.

Next, we asked whether the *pericardin* transcript level is altered in mutant animals and we tested the three alleles *pericardin*^MB03017^, *pericardin*^3-21^, and *pericardin*^3-358^ by FISH (Fig. 2A-F). *pericardin* transcripts were not detectable in homozygous *pericardin*^MB03017^ and *pericardin*^3-358^ animals (Fig. 2B,D), which suggested that the EMS-induced mutation in *pericardin*^3-358^ affects either transcription or transcript stability. By contrast, *pericardin* transcripts were clearly detectable in the allele *pericardin*^3-21^ (Fig. 2F). The latter result indicated an EMS-induced mutation in the *pericardin* locus that does not significantly affect transcription, but rather severely impairs protein functionality (Fig. 1).

Anti-Pericardin staining of embryos or larvae furthermore revealed that in homozygous *pericardin*^3-358^ animals (same as *pericardin*^3-358^/*pericardin*^MB03017^, *pericardin*^3-358^/Df(3L)vin6, and *pericardin*^MB03017^*/pericardin*^MB03017^ individuals) the amount of Pericardin protein is reduced below detection limits (Fig. 2H). By contrast, we found Pericardin being present in homozygous *pericardin*^3-21^ animals (Fig. 2I). As shown previously (Drechsler et al., 2013), localisation of additional ECM constituents such as Nidogen and Laminin was not affected in *pericardin* mutants. This is further corroborated by staining *pericardin*^3-21^ and *pericardin*^3-358^ for Trol/Perlecan (Fig. 2G-I), which showed that Trol is successfully assembled at the cardiac matrix. Western blot analysis (Fig. 2J) confirmed our observation that Prc (mutated form) is present in *pericardin*^3-21^ and absent in homozygous *pericardin*^3-358^ animals. Thus, we consider *pericardin*^3-21^ as a new hypomorphic, and *pericardin*^3-358^ as a new amorphic, *pericardin* allele. Unfortunately, our sequence analysis of the gene region did not result in the identification of the EMS-induced mutations. Due to the fact that the mutant chromosomes display numerous polymorphisms within the *pericardin* locus, it was not possible to differentiate between variation and a real point mutation in an unambiguous manner.

Lack of *pericardin* caused severe cardiac phenotypes (Fig. 1) and eventually results in reduced function or total block of cardiac performance (Drechsler et al., 2013). As shown previously, absence of a functional heart reduces fitness and lifespan but – under laboratory breeding conditions – does not cause immediate death of the fly. In agreement with this previous result, we found that all transheterozygous allelic *pericardin* combinations give rise to viable and fertile adult flies (Table 1).

**Table 1.**
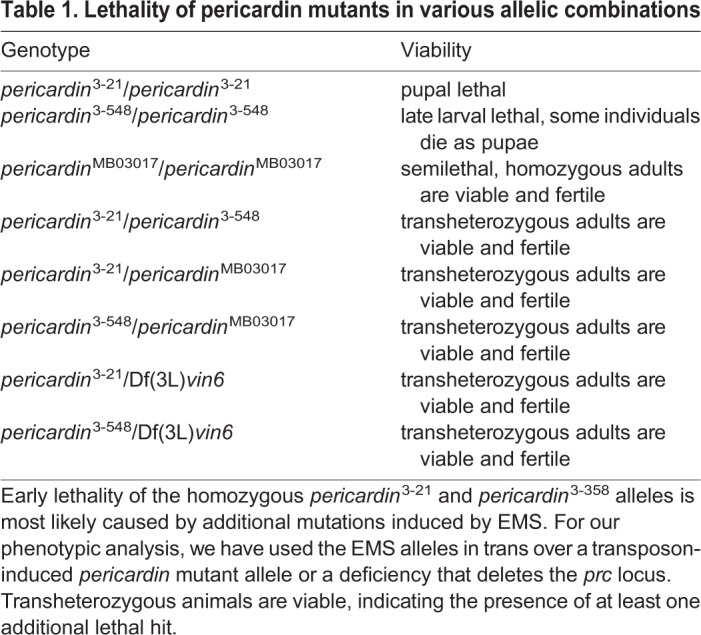
**Lethality of pericardin mutants in various allelic combinations**

### C-terminal GFP fusion proteins mimic endogenous Prc localisation but fail to rescue the *pericardin* phenotype, whereas an untagged version of Pericardin rescues null alleles

**Fig. 3.**
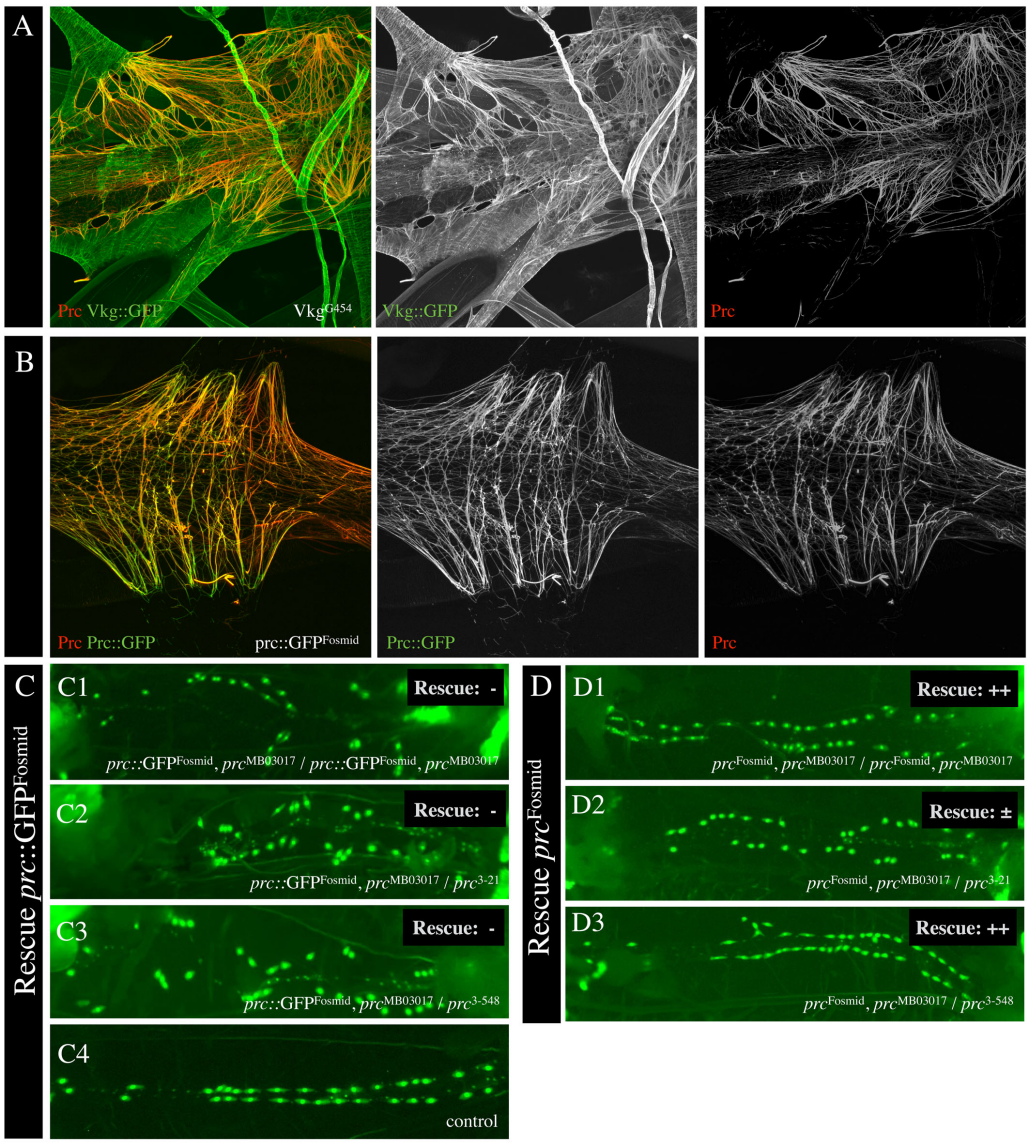
**Prc::GFP localises to the cardiac ECM but fails to rescue the *prc* mutations.** (A) The cardiac extracellular matrix of a dissected third instar wild-type larva (heart chamber region) stained for the Viking::GFP (anti-GFP, green channel) and endogenously expressed Pericardin protein (anti-Prc, red channel). Viking (Collagen IV) stains all basal laminae whereas Pericardin is restricted to the cardiac matrix. (B) The cardiac extracellular matrix of a dissected third instar wild-type larva (heart chamber region) stained for the Pericardin::GFP fusion protein expressed from the fosmid insertion (anti-GFP, green channel) and endogenously expressed Pericardin protein (anti-Prc, red channel). By staining of the endogenous Prc with anti-Prc, the antibody recognises the Prc::GFP protein as well, which indicates complete overlap. (C) Recombinant *prc*::GFP^Fosmid^, *prc*^MB03017^ animals (C1) were mated with *prc*^3-21^ and *prc*^3-548^. F1 larvae at the third instar larval stage were analysed for partial or complete rescue of the cardiac phenotype (dissociation of pericardial cells as a read out for cardiac ECM disassembly). The analysis was carried out in homozygous animals with the genotype *prc*::GFP^Fosmid^, *prc*^MB03017^/*prc*::GFP^Fosmid^, *prc*^MB03017^ (C1) and animals with the genotypes *prc*::GFP^Fosmid^, *prc*^MB03017^/*prc*^3-21^ (C2) and *prc*::GFP^Fosmid^, *prc*^MB03017^/*prc*^3-548^ (C3). (D) *prc*^Fosmid^ was used instead of *prc*::GFP^Fosmid^ for the rescue experiment (D1-D3).

For rescue experiments, we used the fosmid insertion line fTRG497 (*pericardin*::GFP^Fosmid^) (Ejsmont et al., 2009; Sarov et al., 2016). The transgenic fosmid line (fTRG497) duplicates the genomic region 11,945,299 to 11,977,577 that spans the *pericardin* locus. In addition, several epitopes have been fused to the C-terminus of Pericardin including an in-frame sGFP as well as V5, preTEV, BLRP, and 3x FLAG tags. We checked this line for Pericardin production and found that Pericardin is synthesised, secreted (Fig. 3A,B) and incorporates into the ECM of the target tissue (Fig. 3B). The fosmid insertion was recombined with *pericardin*^MB03017^ to generate a *pericardin*::GFP^Fosmid^:: *pericardin*^MB03017^ chromosome. Rescue capability was checked in the homozygous condition and by crossing *pericardin*::GFP^Fosmid^:: *pericardin*^MB03017^ carrying flies to *pericardin*^3-21^ or *pericardin*^3-358^ alleles to generate genetically defined transheterozygous animals with the fosmid rescue construct in the background (Fig. 3C). Presence of the *hand*C-GFP transgene in all genotypes allowed us to stain for the heart and to check for rescue capability (Fig. 3C,D). In addition, we checked various genetic combinations for expression of Pericardin via Western blot analysis (not shown). Animals with the genotype *pericardin*::GFP^Fosmid^, *pericardin*^MB03017^ (Fig. 3C1), *pericardin*::GFP^Fosmid^, *pericardin*^MB03017^/*pericardin*^3-21^ (Fig. 3C2), and *pericardin*::GFP^Fosmid^, *pericardin*^MB03017^/*pericardin*^3-548^ (Fig. 3C3) developed a heart that displayed matrix or disintegration defects, indicating that the Pericardin::GFP encoded by the gene copy present on the fosmid is not able to fully rescue the *pericardin* phenotype. Although the localisation of the *pericardin*::GFP construct appears to be normal, we speculated that the C-terminal tag renders Pericardin non-functional. To test this hypothesis, we generated a new transgenic fly line (*pericardin*^Fosmid^) carrying the identical fosmid at the same landing site as fTRG497, but this time with an untagged version of *pericardin* (fosmid 028051). The VK00033 line (BL9750, Flybase ID FBst0009750) was used for injection, and two independent fly lines were obtained. Line *pericardin*^Fosmid^ was recombined with *pericardin*^MB03017^. The resulting line was used for rescue experiments as described above (Fig. 3D1-D3). We found that – in contrast to the Pericardin::GFP fusion protein – the untagged form of Pericardin rescues the cardiac phenotype of *pericardin* mutants. These experiments confirm that *pericardin*^3-21^ and *pericardin*^3-548^ represent new *pericardin* alleles. The C-terminal GFP-tagged Pericardin fusion protein expressed from the fosmid fTRG497 is properly secreted and assembles into the cardiac matrix, but turned out to be non-functional due to the fused epitope tag.

### Pericardin is highly glycosylated and forms redox-dependent multimers

**Fig. 4.**
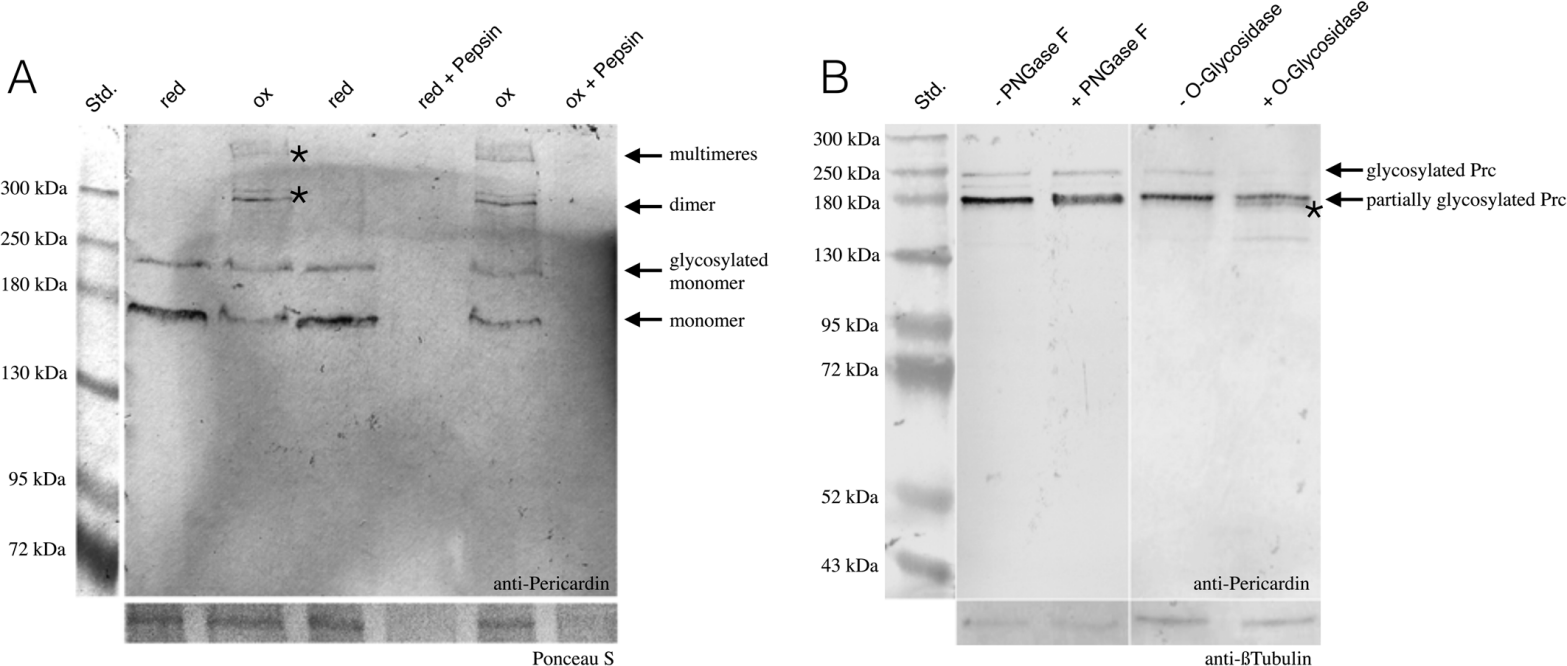
**Mature Pericardin is glycosylated and assembles in redox-dependent multimers.** (A) Protein extracts from wandering third instar larvae (*w*^1118^) were probed with anti-Pericardin antibodies. Under reducing buffer conditions (red), two prominent bands with a molecular mass of approximately 170 kDa and 235 kDa are apparent. Under oxidising conditions (ox), additional high molecular weight bands are detected (asterisks). Pepsin incubation of reduced (red+Pepsin) as well as non-reduced protein preparations (ox+Pepsin) results in absence of any protein detection. (B) Application of PNGase F results in a blurred appearance of the 170 kDa band, concomitant with a slight reduction of the apparent molecular mass. Incubation with O-glycosidase in combination with Neuraminidase results in reduced abundance of the 235 kDa band, which suggests that this band corresponds to fully glycosylated monomeric Prc. In addition, a band slightly below the 170 kDa band appears as a result of O-glycosidase treatment (asterisk).

Pericardin encodes a 1713 aa protein with a predicted molecular mass of 163.5 kDa. Only one single isoform has been described (Chartier et al., 2002; Drechsler et al., 2013). Interestingly, the Pericardin-specific antibody EC11 (Zaffran et al., 1995) detected several bands in protein extracts isolated from wild-type embryos or larvae (Drechsler et al., 2013; Zaffran et al., 1995; this work). To assess the identity of these bands in more detail, we extracted proteins from precisely staged third instar wandering larvae and analysed the defined protein samples under reducing and non-reducing conditions. The defined redox conditions should allow identification of disulfide-bonded multimers, which are characteristic to collagens (Lamberg et al., 1996). As depicted in Fig. 4A, Western blot analysis under reducing conditions with anti-Prc antibodies detected two protein species of approximately 170 kDa and 235 kDa, respectively. Regarding the 170 kDa protein, it appeared likely that this band corresponds to the non-modified monomeric form of Pericardin (predicted molecular mass: 163.5 kDa), whereas the higher migrating protein could represent a glycosylated form of monomeric Pericardin. To address this indication we incubated larval protein extracts with deglycosylating enzymes that are specific to either N-linked (PNGase F) or O-linked glycans (O-glycosidase). Interestingly, application of PNGase F resulted in a blurred appearance of the 170 kDa band, concomitant with a slight reduction of the apparent molecular mass (Fig. 4B). While the blurry appearance may be due to an incomplete deglycosylation, the mass shift clearly indicates that the 170 kDa band corresponds to N-glycosylated monomeric Pericardin. Analysis of the Prc primary sequence identified two putative N-glycosylation sites at positions 72-75 and 1554-1557 (Motif Scan), which suggests a maximum mass shift of 2-3 kDa upon deglycosylation, provided that both predicted sites are functional. The observed shift in molecular mass indicates that at least one of them is glycosylated *in vivo*. Incubation with O-glycosidase in combination with Neuraminidase resulted in a considerable reduction of the 235 kDa band intensity, which suggests that this band corresponds to fully glycosylated Prc, holding both, N- as well as O-linked glycans. In addition, a band slightly below the 170 kDa band appeared as a result of O-glycosidase treatment (Fig. 4B, asterisk), which indicates that also the 170 kDa Prc species holds a limited number of O-linked glycans. Of note, *in silico* analysis predicts 231 O-glycosylation sites within the Prc primary sequence (NetOGlyc 4.0, http://www.cbs.dtu.dk/services/NetOGlyc). The considerable discrepancy between the predicted molecular mass of Prc (163.5 kDa) and the apparent mass of the fully glycosylated protein species (∼235 kDa) indicates that a high portion of the predicted sites are glycosylated *in vivo*. As shown previously, collagens form trimers and these trimers are known to be redox sensitive (Lamberg et al., 1996). Interestingly, we found that also Pericardin forms multimers of high molecular weight (approximately 300 kDa and above 500 kDa) under oxidising conditions, indicating the formation of redox-dependent dimers, trimers, or even higher order multimers, probably stabilised by disulfide bonds (Fig. 4A; Fig. S1). Of note, the formation of disulfide-bonded trimers represents a characteristic feature of all collagens (Fessler et al., 1993). However, unlike collagens, both, monomeric as well as multimeric Prc were susceptible to proteolytic cleavage, as confirmed by Pepsin digestion of reduced and non-reduced protein preparations (Fig. 4A). This result is significant since in most cases multimerisation protects the respective collagen helices from proteolytic digestion (Lamberg et al., 1996). The fact that this protective effect did not apply to Prc multimers may indicate that they are more flexible and thus more accessible to proteases than Collagen IV trimers. Of note, our data suggest that the epitope recognized by the anti-Prc antibody is located within the atypical repeats of the Prc collagen-like domain (Fig. 8). Since this domain is presumably part of the mature triple helix (Chartier et al., 2002), the possibility that only the epitope is cleaved, while the triple helix is still resistant, appears unlikely. We concluded from our analysis that Pericardin is post-translationally modified by glycosylation and that the protein forms redox-dependent dimers and trimers *in vivo*.

### Pericardin modification

Up to now, the biosynthesis and protein maturation pathway of Pericardin has not been well understood. At the sequence level, Pericardin shares a number of conserved sequence motifs with collagens in general, and, in particular, with the three other collagen genes present in the *Drosophila* genome: *Viking*, *Cg25c*, and *Multiplexin* (Volk et al., 2014). Among these motifs present in Pericardin, typical Collagen repeat domains appear to be most characteristic. Collagen repeats are highly enriched in relatively small amino acids, such as glycine, proline, or lysine, which allow the formation of trimers with a compact triple helical structure. Helix formation of Collagen IV, for example, is fostered by hydroxylation of proline and lysine residues, which is mediated by the enzymatic activity of Prolyl4-hydroxylases (PH4) and Lysyl-hydroxylases (LH) in the endoplasmic reticulum (ER). Proline represents the most abundant amino acid in the repeat region of Pericardin and accounts for more than 9% of all amino acids of the full-length protein (lysins, which are also known to be hydroxylated in collagens, account for about 2%). The PH4s in vertebrates and in *Drosophila* consist of α_2_β_2_-tetrameres, with the β-subunit representing the Protein disulfide isomerase (Pdi) (Annunen et al., 1999; Kivirikko and Myllyharju, 1998). In contrast, the Lysyl-hydroxylases form dimers that display enzymatic activity without the necessity of the presence of additional protein-complex components (Kellokumpu et al., 1994). Cross-linking of Collagen or Elastin fibres in the matrix, which ultimately leads to the formation of a ramified meshwork of ECM megafibres, is mediated by the extracellular enzymatic activity of Lysyl oxidases (Lox) (Kagan and Li, 2003). Two *lox* genes are present in the *Drosophila* genome (Molnar et al., 2005), and it has been shown that they regulate ECM stiffness in the nervous system of the fly (Kim et al., 2014). Thus, we tested whether one or more of the known enzymatic activities involved in Collagen biosynthesis and processing, such as PH4, LH or Pdi play a major role in Pericardin processing.

#### Pdi

**Fig. 5.**
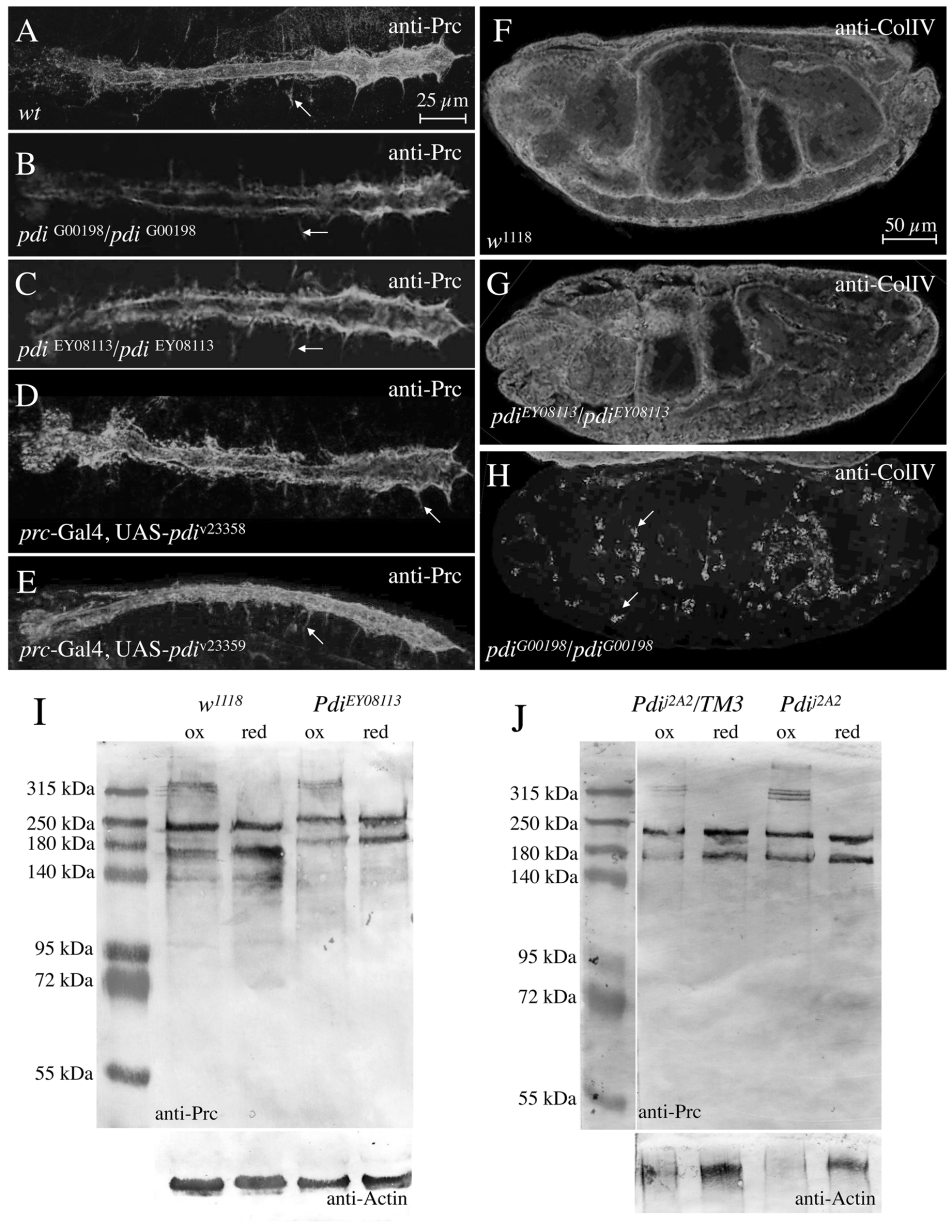
**Pdi has no visible effect** **on Pericardin (Prc) deposition or multimerisation.** (A-H) We tested the potential role of Pdi on Prc secretion and deposition using *pdi* mutants stained for anti-Prc (A-E) or anti-ColIV (F-H). (I-J) To verify a potential role of Pdi on Prc multimerisation, we extracted protein from control and *pdi* mutants and probed the extracts by Western blot analysis. We tested RNAi-mediated down-regulation of *pdi* in adipocytes and pericardial cells, the main resources for structural ECM proteins (D,E). RNAi constructs (v23358 and v23359) were driven by *prc*-Gal4. Protein extracts isolated from *Pdi*^EY08113^ (I) and *Pdi*^j2A2^ (J) specimens were probed under oxidising and reducing conditions. Arrows in A-E highlight Pericardin-positive segmental ECM structures (compare to Fig. 6C). Arrows in H indicate exemplary hemocytes accumulating ColIV.

First, we analysed whether the Protein disulfide isomerase in *Drosophila*, encoded by the single gene *pdi* (*dpdi*), plays a role in cardiac matrix formation and Pericardin processing (Fig. 5). We tested three classical *Pdi* alleles and two RNAi lines. Two alleles, *pdi*^G00198^ and *pdi*^J2A2^, were induced by the insertion of transposons, which are located at very similar positions within the first intron of *Pdi* (Flybase, 1999; Morin et al., 2001; Spradling et al., 1999). Both alleles are described as being capable of inducing lethality. The third allele, *pdi^EY^*^08113^, caused by a P-insertion in the 5′prime UTR region of *pdi*, is homozygous viable, and thus can be considered as a potentially hypomorphic allele (Bellen et al., 2004). Neither the three alleles induced by P-element insertions nor the two RNAi-mediated down-regulation instances of *pdi*, in which we used *prc-*Gal4 for inducing expression of the respective RNAi construct (v23358, Fig. 5D and v23359, Fig. 5E), caused severe abnormalities with respect to Pericardin deposition at the embryonic cardiac matrix (Fig. 5A-E). As control, we stained for Collagen IV (Fig. 5F-H), which is thought to be retained in collagen-producing cells in strong *pdi* mutants. Indeed, in contrast to wild type (Fig. 5A) or the hypomorphic allele *pdi^EY^*^08113^ (Fig. 5G), Collagen IV was retained in *pdi^G00198^* embryonic haemocytes (Fig. 5H). Western blot analysis of proteins isolated from hypomorphic or amorphic embryos (Fig. 5I,J) confirmed presence of Pericardin in its monomeric, glycosylated, and multimeric forms in all cases tested.

**Fig. 6.**
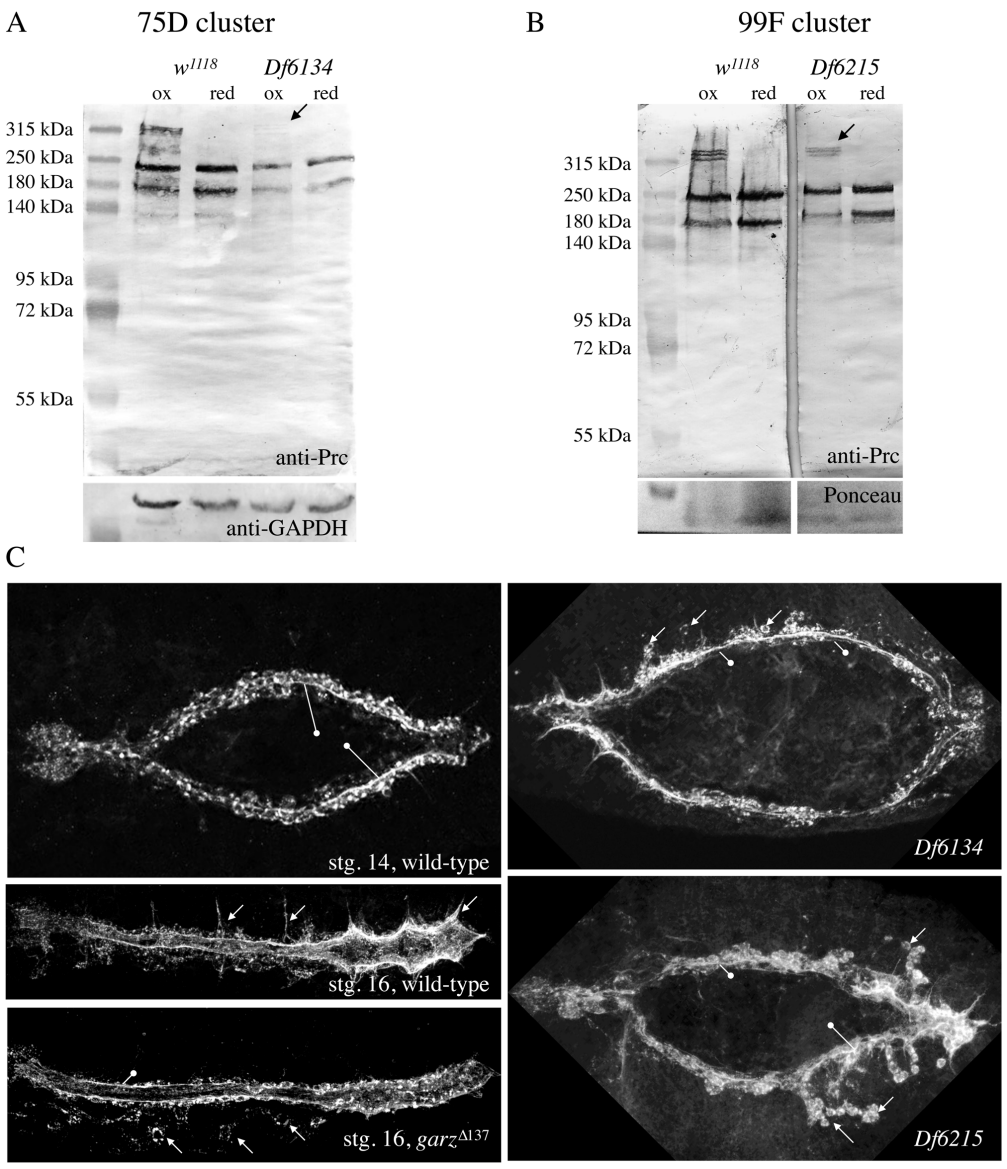
**Deletion of the PH4 gene cluster at 75D inhibits efficient higher-order multimerisation of Pericardin.** (A,B) Protein extracts isolated from homozygous animals with the genotype Df(3L)Exel6134 (deleting the PH4 gene cluster at 75D) (A) and Df(3R)Exel6215 (deleting the PH4 gene cluster at 99F) (B) were probed for anti-Pericardin under oxidising and reducing conditions. As loading control Western blots were probed for GAPDH or membranes were stained for Ponceau. (C) Wild-type, homozygous Df(3L)Exel6134 and Df(3R)Exel6215 and homozygous *garz*Δ^137^ mutant embryos (stg. 16/17) were stained with anti-Prc.

#### Possible role of Prolyl-hydroxylases for cardiac ECM assembly and maintenance

At least 19 genes encoding Prolyl4-hydroxylase α-subunits are present in the *Drosophila* genome; ten map to 99F8-9, six map to a cluster at 75D3-4, and three are dispersed to other chromosomal regions (Abrams and Andrew, 2002). Together with Pdi, they form the enzymatically active tetrameric Prolyl-hydroxylase complex, processing collagens in the ER of the cell (Annunen et al., 1999). Whether the PH4 proteins display a rigid substrate specificity or whether they process a broader spectrum of target proteins in a tissue-specific manner is yet unknown. However, it has been shown previously that one of the PH4 proteins at 99F, PH4αEFB (CG31022), affects *Drosophila* Collagen IV (Viking) processing efficiently. Thereby, PH4αEFB RNAi-mediated knock-down in adipocytes results in larvae in which Viking is secreted in its monomeric form. Under these conditions, Viking accumulates in the haemolymph but fails to become incorporated into BMs (Pastor-Pareja and Xu, 2011). Therefore, we tested PH4αEFB mutants as well as PH4αEFB RNAi-mediated knock-down in adipocytes for a possible influence on Pericardin secretion and multimerisation. It should be noted that PH4αEFB is the only PH4 protein, according to the expression data published in FlyAtlas and elsewhere, which is strongly co-expressed with Pericardin in larval adipocytes. In any case, Pericardin production appeared normal and unaltered under the experimental conditions we selected (data not shown). Therefore, we tested deficiencies that remove several Prolyl4-hydroxylase α-subunits at the same time, for instance the PH4 clusters at 75D and 99F with six and ten PH4 genes, respectively (Fig. 6). The deficiency Df(3L)Exel6134 deletes 16 genes including all six PH4 genes at 75D3 (Fig. 6A). The ten Prolyl4-hydroxylases clustered at 99F are removed by the deficiency Df(3R)Exel6215, which removes 15 genes in total (Fig. 6B). Homozygous mutants die during embryogenesis at stage 14, or 15 at the latest, before heart formation is completed and a ramified pericardin network is formed (Fig. 6C). However, we found that Prc is synthesized and secreted as indicated by the formation of Prc sheets and layers around the malformed heart tube in both mutants. For comparison we present *garz*^Δ137^ mutant embryos stained for Pericardin (Fig. 6C). It has been shown previously that secretion of Pericardin protein is inhibited in garz mutant embryos (Wang et al., 2012). However, our Western blot analysis of wild-type and PH4 mutants indicated a possible effects on Pericardin multimerisation (Fig. 6A,B). The upper-most band, corresponding to Prc multimeres, is severely reduced in homozygous Df(3L)Exel6134 mutant embryos (deleting the 75D PH4 gene cluster) and slightly reduced in homozygous Df(3R)Exel6215 mutant embryos (deleting the 99F PH4 gene cluster) (Fig. 6A,B). Testing individual PH4 genes from the 75D cluster by utilising RNAi-mediated down-regulation did not result in the identification of one single PH4 protein responsible for Pericardin processing. Possible explanations might be the insufficient efficiency of RNAi-mediated down-regulation of the respective genes or a redundant functionality of PH4s from the cluster, an issue that needs to be analysed further in future studies. However, our results indicated that hydroxylation of Pericardin might take place and insufficiently hydroxylated forms of Pericardin might fail to form multimers. It has been shown for vertebrate collagens that less hydroxylated proteins are less stable than the fully processed proteins and become degraded by the cell, a hypothesis that is corroborated by data on other collagens, which are degraded in the absence of ascorbic acid (vitamin C), a key cofactor of PH4 in mammals (Murad et al., 1981), as well as the fact that lack of functional PH4 keeps collagens retained in the ER of collagen-producing cells (Walmsley et al., 1999). Whether this applies to Pericardin as well remains to be elucidated in future experiments.

#### Lysyl-hydroxylase (dPlod)

**Fig. 7.**
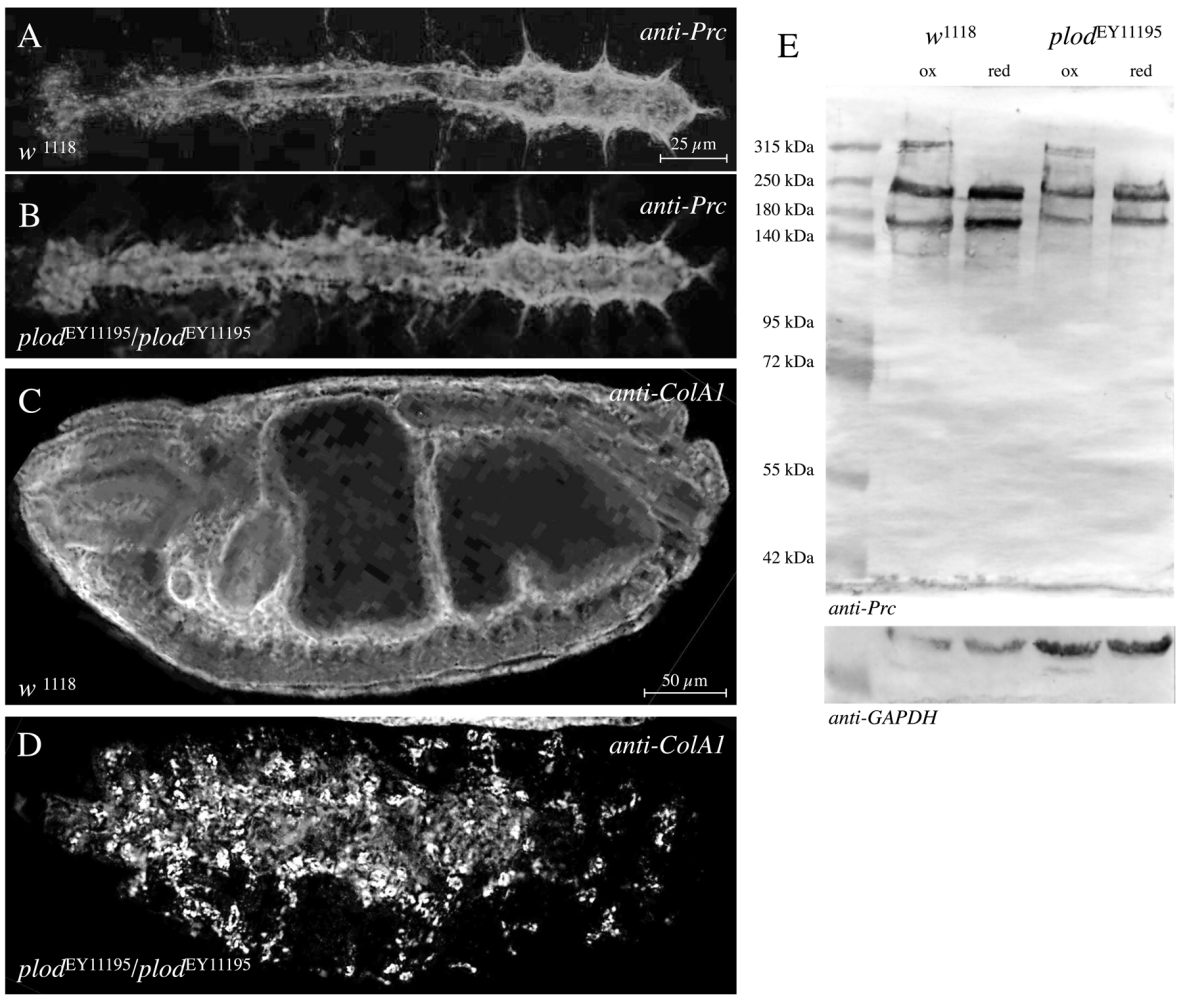
**Plod has no visible effect on Pericardin deposition or multimerisation.** (A-D) We tested the potential role of dPlod on Pericardin secretion and deposition using the molecularly characterised *dplod* mutant allele *dplod*^EY11195^ (B,D). Stage 17 embryos were stained for anti-Prc (A,B). The same allele was probed for anti-ColIV (C,D). (E) To check for a potential role of Plod on Pericardin multimerisation, we extracted protein from control and *dPlod* mutants and probed the extracts by Western blot analysis. As a loading control Western blots were probed for GAPDH.

The *Drosophila* genome harbours a single orthologue of the vertebrate Lysyl hydroxylase 3 named dPlod (Bunt et al., 2011, 2010). This enzyme catalyses hydroxylation of lysine residues in collagens. In agreement with its expected function, *dPlod* is highly expressed in type-IV Collagen-producing cells that are haemocytes and adipocytes. According to FlyAtlas, *dPlod* shows an even broader tissue and temporal expression including embryonic and larval heart and fat body tissue; thereby, *dPlod* is also co-expressed with *pericardin*. Bunt and colleagues showed that Collagen IV is retained in embryonic haemocytes in *dPlod* mutants using a deficiency that removes the *dPlod* locus and eight additional genes (Bunt et al., 2011). Animals carrying the deficiency die during embryogenesis when homozygous for the deletion. Until now, FlyBase has annotated six P-element insertions associated with the *dPlod* locus; all of them locate within the 5′prime UTR of *dPlod* and none of them cause lethality (Flybase, 1999). We tested one of the published lines, *dPlod*^EY11195^, for a possible impact on Pericardin biosynthesis and found no distinct effect on the secretion of Pericardin or its incorporation into the embryonic cardiac ECM (Fig. 7A,B). Western blot analysis confirmed that multimerisation of Pericardin is not affected in this *dPlod* allele when compared to wild-type (Fig. 7E). As control, we used the identical line to stain for Collagen IV (Fig. 7C,D). We observed that Collagen IV is retained in the embryonic haemocytes, a result that confirmed previously made observations (Bunt et al., 2011) and also showed that this gene-specific P-insertion affects dPlod function. Our result indicates that Lysyl-hydroxylation plays no major role in Pericardin biosynthesis and maturation.

### Expression of Pericardin in H5 cells

**Fig. 8.**
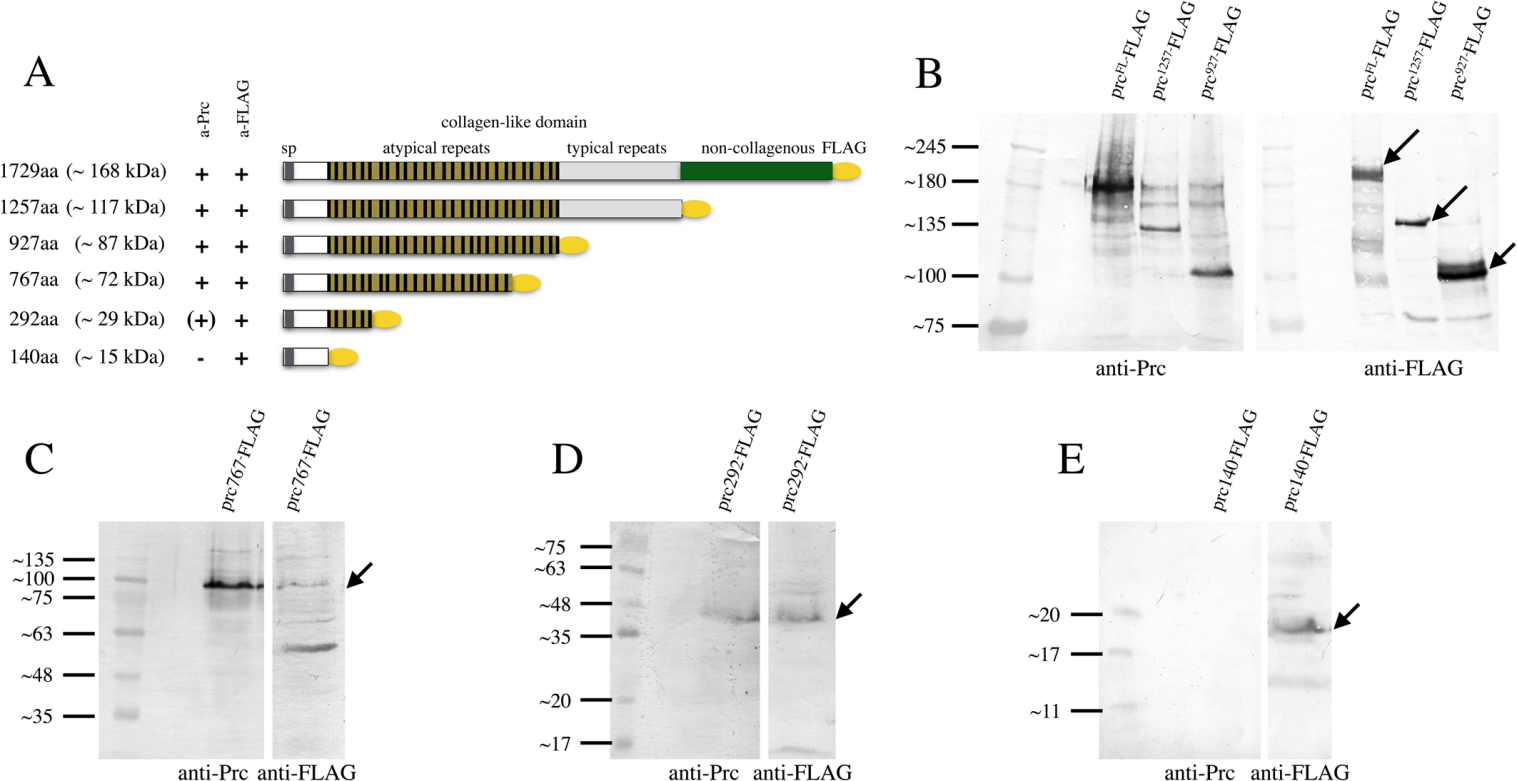
**Expression of Pericardin constructs in H5 cells uncovers the epitope binding region of EC11 antibody.** (A) Graphical presentation illustrating the FLAG-tagged Prc constructs used for expression in H5-cells. (B-E) A series of C-terminal deletions was generated and expressed, and protein extracts from H5 cell cultures were probed each for anti-Prc and for anti-FLAG.

To produce larger amounts of the protein in cultured cells for future biochemical and structural characterisation, we checked for possible expression of Pericardin in High-Five (H5) cells. Furthermore, we considered a cell culture system for expressing Pericardin as being potentially helpful to elucidate the function of protein domains present in the primary sequence of Pericardin, such as non-typical Collagen IV repeats or one RGD site close to the C-terminus of the protein. The latter motif might account for anchoring Pericardin to Integrin receptors at the cell surface. Therefore, we induced expression of a full-length FLAG-tagged version of Pericardin in H5 cells (Fig. 8). Interestingly, we found that H5 cells synthesise predominantly the monomeric, partially glycosylated form of Pericardin (Fig. 8B), whereas the fully glycosylated monomer or dimers and trimers are almost undetectable. Of note, earlier experiments that aimed to express human collagens in H5 cells demonstrated that multimerisation of the proteins fails due to the absence of the appropriate PH4 enzymes in the cultured cells (Lamberg et al., 1996). In line with these data, we have shown that a yet unknown PH4 (or a combination of several) at cluster 75D seems to be important, at least to some degree, to proper Prc multimer formation (Fig. 6A). Thus, lack of proper PH4 enzymes in H5 cells might explain the absence of higher-order multimers of Pericardin when expressed in this cell type.

Nevertheless, we used our full-length FLAG-tagged Pericardin construct as a starting plasmid to generate a series of truncated versions and expressed all of them in H5 cells to map the epitope recognised by EC11 (Fig. 8A). Since the antibody detects both monomeric, as well as multimeric Pericardin, lack of proper multimerisation is considered insignificant in this context. Our Western blot analysis showed that the EC11 antibody binds to an epitope within the N-terminal third of Pericardin, mapped between amino acids 140 to 292. Our data are in conflict with a previous report that showed that the EC11 antibody recognises a short sequence at the C-terminus of Pericardin (Chartier et al., 2002). The authors of the respective study used a trypsin-digested ECM preparation that was probed with EC11 as a basis for immuno-precipitation. Subsequently, a Pericardin antigen peptide with the sequence NFQSTYYTK was purified. This sequence is identical to a short stretch located at the C-terminus of Pericardin. However, the EC11 anti-Pericardin antibody clearly recognises Pericardin forms with truncations at the C-terminus (Fig. 8); therefore, we speculate that the previous results may have been impaired by off-target binding of the antibody.

## DISCUSSION

The ECM protein Pericardin plays a fundamental role in supporting the structural integrity of the cardiac matrix in the developing *Drosophila* embryo and larvae (Chartier et al., 2002; Drechsler et al., 2013). Lack of Pericardin or inhibition of Pericardin recruitment to the cardiac matrix results in destabilisation of the larval cardiac ECM meshwork and loss of the alary muscles-pericardial nephrocytes-heart tube connection upon initiation of heart beat activity. Finally, upon ageing, this leads to luminal heart collapse and renders the heart nonfunctional (Fig. 1). Here, we introduce two new EMS-induced *pericardin* mutants that both display the characteristic cardiac phenotypes; one of the alleles turned out to be a null allele characterised by complete absence of the protein (Fig. 2). All *pericardin* alleles and transheterozygous combinations of *pericardin* alleles not only show ECM disintegration upon ageing but also heart collapse associated with disorientation of the cardiomyocyte sarcomeres. In wild-type animals the sarcomeres are highly organised and show a helical orientation (Lammers et al., 2017; Lehmacher et al., 2012). In *pericardin* mutants this orientation is lost, presumably due to the fact that the costameres lose their link – via integrins – to the extracellular matrix upon ECM disintegration.

Pericardin co-localises with type IV Collagen (Viking) (Fig. 3A). However, in contrast to Viking, which assembles into the basal lamina of virtually all tissues within the animal, Pericardin is highly restricted to the cardiac ECM. ECM fibres harbouring Viking and Pericardin connect the heart tube to the alary muscles (Fig. 3A). A Pericardin::GFP fusion protein expressed from an engineered fosmid carrying an approximately 40 kb genomic region including the *pericardin* locus with the *pericardin* gene tagged with GFP, is synthesised, secreted, distributed by haemolymph flow and assembles at the cardiac matrix (Fig. 3B). Co-staining for Prc::GFP and endogenous Pericardin shows a complete overlap. However, the Prc::GFP fusion protein fails to rescue the cardiac phenotype of *pericardin* mutants (Fig. 3C). By contrast, Pericardin, expressed from an identical fosmid but lacking the C-terminal GFP tag, harbours rescue capability (Fig. 3D). This demonstrates the importance of the C-terminus of Pericardin for full functionality. While future studies are needed to analyse why Prc::GFP fails to rescue, it appears likely that the C-terminal tag affects accessibility of the RGD-site, which is located close to the C-terminus and which might play a role in anchoring Pericardin to the cell surface via Integrin interaction.

Based on distinct sequence similarities, including a central Collagen-like repeat domain with typical (Gly-X-Y)*_n_* repeats, Pericardin was classified as a type IV Collagen-like protein. In addition, it has been speculated that, analogous to collagens, Pericardin has the ability to form triple helices (Chartier et al., 2002). However, experimental evidence for dimer-, trimer- or multimerisation of Pericardin has not been provided yet. By analysing protein samples under defined redox conditions, we found that non-reducing conditions result in formation of high molecular weight Prc multimers. Based on the apparent molecular mass (>500 kDa), the largest multimers most likely correspond to trimeric or even higher order multimeric Prc (Fig. 4A; Fig. S1). Thus, similar to collagens (Lamberg et al., 1996), Prc appears to form redox-dependent multimers, probably disulfide-bonded. Considering the fact that Prc is embedded into the cardiac extracellular matrix, which resides in an oxidising environment, multimeric Prc likely represent the mature, functional form, while the monomeric species presumably constitute biosynthesis intermediates. In addition to confirming Prc multimerisation, we also found that the protein is extensively glycosylated. Application of both, N- as well as O-glycosidic bond-specific enzymes resulted in distinct mass shifts (Fig. 4B). While the apparent shift of about 2-3 kDa resulting from PNGase F incubation suggests presence of 1-2 N-linked glycans, the huge mass shift that is obvious upon O-glycosidase incubation (∼65 kDa) indicates substantial O-glycosylation of the protein in the Golgi. This indication is supported by sequence analysis, which predicts only two N-glycosylation (MotifScan) but 231 O-glycosylation sites (NetOGlyc 4.0). Glycosylation represents a highly prevalent post-translational modification of ECM proteins and accounts for cell-cell and cell-matrix attachment by promoting the formation of ramified networks between the glycosylated proteins present in the matrix. Considering this, as well as the severe effects of Prc knock out (Drechsler et al., 2013), glycosylated Pericardin appears to be a core component of the ECM network present at the heart.

Trimerisation of type IV Collagen has been shown to depend on the enzymatic activity of Prolyl (PH4)- and Lysyl (LH)-Hydroxylases. Prolyl-Hydroxylases form a tetrameric complex, with Proteindisulfide-Isomerase (Pdi) being present in the complex. Hydroxylation occurs in the Collagen producing cells in the lumen of the ER prior to secretion of Collagen IV molecules (Stephens, 2012). Interestingly, the primary sequence of Pericardin contains a high number of prolines (158, 9.2% of total) as well as numerous type IV Collagen-like repeats, which indicates that Pericardin may undergo a similar biosynthesis pathway as collagens. Therefore, we analysed whether formation of high molecular mass forms of Pericardin (dimers and trimers) is affected in Pdi (Fig. 5), PH4 (Fig. 6) or LH (Fig. 7) mutants. Only in PH4 mutants we observed the absence of these multimers in Western blot analysis. The PH4 mutants we used harbour deletions that remove six annotated Prolyl-Hydroxylases at once, the so-called 75D cluster. Single mutant lines for each of these PH4s are not available; therefore we tested whether RNAi-mediated down-regulation of the individual PH4 genes in the cluster results in an inhibition of the formation of high molecular mass forms of Pericardin, which was not the case. This is either caused by inefficient down-regulation of the target gene or by a redundant function of more than one of the PH4s in the 75D cluster. Pdi and LH seem to play no major role in Pericardin biosynthesis (Figs 5 and 7).

In H5 cells, which are widely used to express high amounts of recombinant protein for further biochemical characterisation, Pericardin is not expressed endogenously at detectable levels (data not shown). Using a full-length FLAG-tagged version of Pericardin we found that, after the cells were transfected with the construct, only the monomeric form of Pericardin is produced (Fig. 8). We conclude that H5 cells derived from the cabbage looper *Trichoplusia ni* lack activity of certain enzymes critical to the biosynthesis of multimeric forms of Pericardin. Interestingly, it has been noticed earlier that the expression of human Collagens is highly efficient in H5 cells, but multimerisation fails due to absence of the appropriate PH4 enzymes in the cultured cells (Lamberg et al., 1996). Our result that a yet unidentified PH, or a combination of several PHs from the 75D cluster, appears to be essential to proper formation of Pericardin multimers indicates that, like in Collagens, Pericardin multimer formation fails due to absence of the required PH in the H5 cells. However, we successfully used the cell culture system to determine the epitope recognised by the widely used EC11 antibody. EC11 recognises Pericardin in the native and the denatured state and our experiments indicate that the epitope bound by this antibody locates to the N-terminus of Pericardin (Fig. 8).

## MATERIALS AND METHODS

### Drosophila stocks

Fly strains used in this study were *w*^1118^ *vkg*::GFP-454 (Collagen IV α2) (Morin et al., 2001), and *hand*C-GFP (Hallier et al., 2015; Paululat and Heinisch, 2012; Sellin et al., 2006). The following strains were obtained from the Bloomington *Drosophila* Stock Center: *pdi*^G00198^ (BL110624, FlyBase ID FBst0307666), *pdi*^EY08113^ (BL19868, FlyBase ID FBst0019868), *pdi*^j2a2^ (BL12090, FlyBase ID FBst0012090) *dplod*^EY11195^ (BL20273, FlyBase ID FBst0020273), Df(3R)6134 (BL7613, FlyBase ID FBst0007613) (deletes the PH4 cluster at 75D1), and Df(3R)Exel6215 (BL7693, FlyBase ID FBst0007693) (deletes the PH4 cluster at 99F8). The RNAi lines v23358 (FlyBase ID FBst0454967) and v23359 (FlyBase ID FBst0454968) were obtained from the Vienna Stock Collection.

### Mapping of PMM3-21 and PMM3-548

To map the mutation, we generated transheterozygous flies with PMM3-21 and PMM3-548, and approximately 160 deficiency lines from the third chromosome deficiency kit (FlyBase, Bloomington, USA), and scored the progeny for the presence of the cardiac phenotype. Subsequently, fine mapping was performed with smaller deletions, transposon and EMS mutants that affect individual genes.

### Rescue of prc-mutants

For rescue experiments, we used the fosmid line fTRG947 (*pericardin*::GFP^Fosmid^) (Ejsmont et al., 2009; Sarov et al., 2016) (gift from F. Schnorrer, IBDM - Institute de Biologie du Developpement de Marseille, Marseille, France) and *pericardin*^Fosmid^, which duplicate the genome region 11,945,299 to 11,977,577. Both lines were recombined with pericardin^MB03017^ and used in trans to *pericardin*^3-21^ and *pericardin*^3-548^. For generating transgenic *Drosophila* lines, a commercial service was used (BestGene, Chino Hills, USA). The *Drosophila* line carrying the landing side was BL9750 (FlyBase ID FBst0009750).

### Immunohistochemistry

Wandering third instar larvae were dissected and stained as previously described (Drechsler et al., 2013). The following antibodies were used: rabbit anti-Nidogen (1:1000, gift from S. Baumgartner, Faculty of Medicine/Department of Experimental Medical Science, Lund University, Lund, Sweden), mouse anti-Pericardin [1:5, Developmental Studies Hybridoma Bank (DSHB), Iowa City, USA], rabbit anti-Perlecan/Trol (1:1000, gift from S. Baumgartner), mouse anti-Col4A1 (1:50, gift from M. Mink, Department of Genetics, University of Szeged, Szeged, Hungary) and rabbit anti-GFP (1:2000, Abcam). Secondary antibodies used were anti-mouse Cy2/Cy5 and anti-rabbit Cy3/Cy5 (1:100, Dianova /Abcam).

### Deglycosylation assay

Animals were collected and anaesthetised on ice. Per reaction, five wandering third instar larvae were homogenised in 100 µl Glycoprotein Denaturing Buffer (0.5% SDS, 40 mM DTT) and boiled for 10 min. PNGase F and O-Glycosidase/Neuraminidase treatments (12 h, 37°C) were done according to the manufacturer′s instructions (New England Biolabs, Ipswich, USA). Subsequently, samples were boiled in Laemmli buffer and subjected to SDS-PAGE and Western blot analysis.

### H5 cells, transfection, and protein extraction from H5 cells

Cell culture and transfection was performed as described in (Hallier et al., 2016). For heterologous expression in H5 cells, full-length Prc was cloned into the pFastBacDual vector (Life Technologies). The endogenous stop codon of Prc was removed and a C-terminal FLAG tag was inserted by appropriate primer design. Truncated versions of Pericardin were established using the respective FLAG tagged construct as a template.

## Supplementary Material

Supplementary information
