# Supplementary material for: Biosynthesis and assembly of the Collagen IV-like protein Pericardin in Drosophila melanogaster
Source: Biol Open. 2018 Apr 15;7(4):bio030361. doi: 10.1242/bio.030361 (PMC5936059; doi:10.1242/bio.030361)
Supplement: Supplementary information [file biolopen-7-030361-s1.pdf]

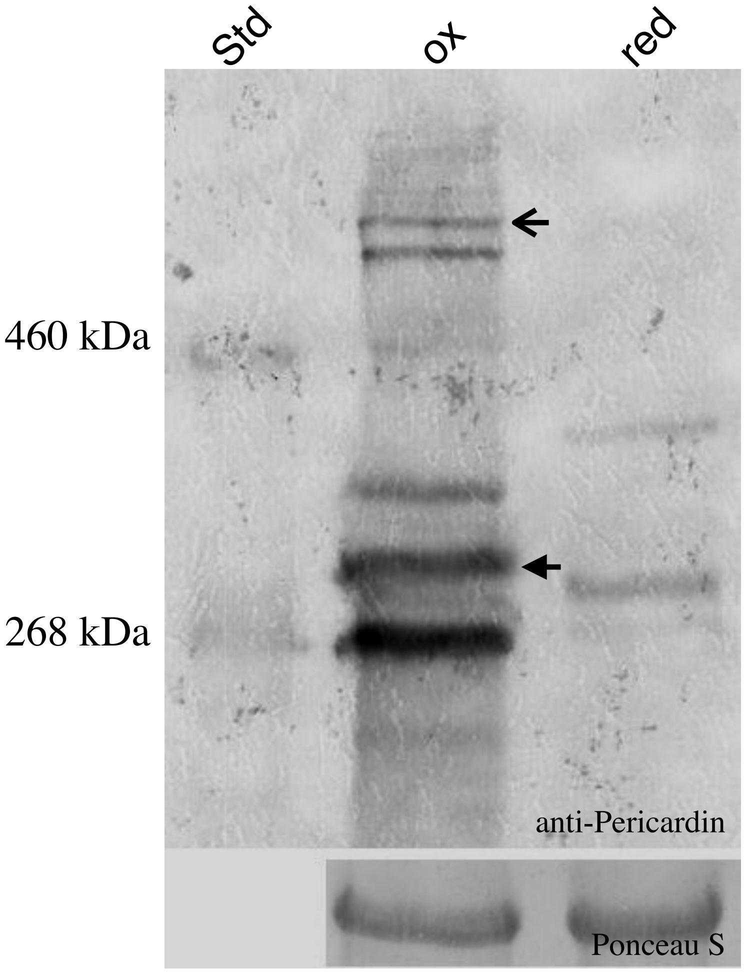

Fig. S1 Mature Pericardin assembles in redox-dependent di- and trimers

Total protein extracts from wandering 3rd instar larvae (w^{1118}) were separated by high-resolution (8%) SDS-PAGE, transferred to nitrocellulose membranes, and probed with anti-Pericardin antibodies. Under oxidising conditions (ox), high molecular weight bands are detected above 300 kDa (arrow) and above 500 kDa (open arrow), which are absent under reducing (red) conditions.
